# Circulating Tumor Cells: Application as a Biomarker for Molecular Characterization and Predictor of Survival in an All-Comer Solid Tumor Phase I Clinical Study

**DOI:** 10.1371/journal.pone.0058557

**Published:** 2013-08-21

**Authors:** Haifeng Bao, Patricia A. Burke, Jiaqi Huang, Xiaoru Chen, Philip Z. Brohawn, Yihong Yao, Robert J. Lechleider, Robert S. Sikorski, Manuela Buzoianu, Jianliang Zhang, Xiaoqing Shi, Laura K. Richman, Theresa M. LaVallee

**Affiliations:** 1 Department of Translational Sciences, MedImmune LLC, Gaithersburg, Maryland, United States of America; 2 Formerly at Clinical Development, MedImmune LLC, Gaithersburg, Maryland, United States of America; 3 Clinical Development, MedImmune LLC, Gaithersburg, Maryland, United States of America; Queen Elizabeth Hospital, Hong Kong

## Abstract

**Purpose:**

Clinical development of cancer drugs has a low success rate. Prognostic and predictive biomarkers using minimally invasive approaches hold promise for increasing the probability of success by enabling disease characterization, patient selection and early detection of drug treatment effect. Enumeration and molecular characterization of circulating tumor cells (CTC) may address some of these needs, and thus were evaluated for utility in a Phase I solid tumor clinical study.

**Experimental Design:**

Blood samples for CTC analysis were obtained from 24 cancer patients in a multi-center all-comer Phase I study of MEDI-575, a novel anti-PDGFRα antibody. Samples were taken at screening and analyzed for enumeration of CTC using the CellSearch^®^ platform and for molecular characterization using a novel quantitative RT-PCR assay.

**Results:**

Fifty-nine percent of the patients showed at least 1 CTC per 7.5 ml of blood at baseline. Progression-free survival (PFS) and overall survival (OS) of patients with 0 CTCs at baseline were longer than PFS and Os for patients with 1-3 and >3 CTCs (8.8 versus 1.4 and 1.3 months PFS, *P* = 0.02; 9.0 vs 7.4 and 3.5 months OS, P = 0.20, respectively). Patients with 0 CTC showed a greater percentage of stable disease than the other 2 groups with 1-3 and >3 CTCs (57% vs 29% and 0%). The multimarker qRT-PCR method detected CTC in 40% of the patients, and 80% of these patients were positive for pre-selected drug target genes.

**Conclusion:**

CTC enumeration of patients in an all-comer study is feasible and may allow for patient stratification for PFS and Os to evaluate the clinical response of investigational agents. Gene expression profiling of isolated CTC may provide a means for molecular characterization of selected tumor targets.

## Introduction

Circulating tumor cells (CTC) have been found in the peripheral blood of patients with a wide range of solid tumors such as breast, colorectal, lung, prostate, ovarian, pancreatic, liver, and bladder [1,2]. Detection of CTC has been shown to be a strong predictor of poor progression-free and overall survival of patients with metastatic disease [3–5]. Emerging evidence from clinical studies also demonstrates that changes in a patient’s CTC count after treatment may indicate the effectiveness of a therapeutic intervention [6–8]. CTC may have many of the molecular characteristics of the primary tumors and metastases and reflect changes in the phenotype and genotype of the tumor cells taking place after the original diagnosis or tumor excision [9,10]. Therefore, CTC analysis, including enumeration and molecular characterization, holds great potential to provide a method for the real-time monitoring of disease progression and therapy response as well as to stratify patients most likely to respond to a given targeted therapy.

The transmembrane receptor tyrosine kinase platelet-derived growth factor receptor-α (PDGFRα) plays an important role in human carcinogenesis, both as a direct target on tumor cells and as a mediator of stromal support for cancer cell growth. Expression of PDGFRα has been observed in multiple solid tumors, including lung [11], breast [12], prostate, ovarian, and hepatocellular carcinomas [13]. MEDI-575 is a human IgG2 antibody with high affinity and specificity for human PDGFRα. In preclinical studies, therefore, could potentially reduce the growth of solid tumors. The Phase I clinical study results reporting safety and pharmacokinetics in the weekly dose escalation portion of the study have been previously reported [14].

We assessed both the feasibility of performing and the utility of CTC analysis as a potential biomarker in the multicenter Phase I clinical trial of MEDI-575. We used the FDA-approved CellSearch^®^ CTC test to analyze the frequency of CTC in subjects with solid tumors treated with MEDI-575. We also developed a multimarker qRT-PCR assay to assess molecular characteristics of CTC

## Materials and Methods

All patient samples were obtained as part of the Phase I clinical trial that is listed at http://clinicaltrials.gov/ct2/show/NCT00816400. All samples were collected and analyzed with written informed consent using a protocol approved by the US Oncology Institutional Review Board. open-label, dose-escalation, Phase I clinical trial of MEDI-575 between March 2009 and January 2011. MEDI-575 was administered by intravenous infusion once weekly for 3 weeks at doses of 3, 6, 9, 12, and 15 mg/kg and every 3 weeks at doses of 25 and 35 mg/kg in this dose escalation study. A total of 7.5 ml of blood were drawn into CellSave® tubes (Veridex LLC) at patient screening and at Day 1 pre-perfusion of Cycle 2 and subsequent cycles for CTC enumeration. An additional 7.5 ml of blood was collected at screening for gene expression profiling.

### CellSearch® CTC enumeration

Blood samples collected in CellSave® tubes were maintained at room temperature and processed within 96 hours of collection. CTC isolation and enumeration were conducted using the CellSearch® System (Veridex LLC) at MedImmune as described previously [15]. Briefly, 7.5 ml of blood were gently mixed with 6.5 ml of dilution buffer, centrifuged for 10 minutes at 800 x g at room temperature, and transferred onto the CellTracks® AutoPrep system. After aspiration of the plasma and dilution buffer layer, anti-EpCAM antibody-coated ferrofluids were added. After immunomagnetic separation, enriched cells were permeabilized and then fluorescently labeled with FITC-labeled antibodies recognizing cytokeratins 8, 18, 19 and allophycocynanin-labeled antibodies recognizing CD45. After incubation on the system, the magnetic separation was repeated and excess staining reagents were aspirated, and cell nuclei were stained with the nucleic acid dye 4’, 6’-diamidino-2-phenylindole (DAPI). The sample was transferred automatically to a cartridge in the MagNest® Cell presentation Device. Identification and enumeration of CTC was performed using the CellTracks® Analyzer II, a semiautomated fluorescence microcopy system that permits computer-generated reconstruction of cellular images. CTC were defined as nucleated cells lacking CD45 and expressing cytokeratin and were enumerated by trained operators.

### Multimarker quantitative RT-PCR assay

Blood samples collected in CellSave® tubes were maintained at room temperature and processed within 96 hours of collection. CTC enrichment was performed using the CellSearch Profile Kit (Veridex LLC). RNA was isolated from the resulting CTC-enriched samples, and then cDNA was generated utilizing the SuperScript III kit (Invitrogen) following the manufacturer’s random hexamer priming protocol. The cDNA samples were cleaned and concentrated utilizing the Agencourt RNAClean bead reagent (Beckman Coulter Genomics.), followed by a pre-amplification reaction using the Applied Biosystems (ABI). The resulting reaction mix was analyzed for defined target genes using Applied Biosystems 20X Taqman assays on the Fluidigm Biomark 48.48 Dynamic Array according to the manufacturer’s protocol. Initial data analysis was carried out utilizing the Fluidigm Gene Expression Analysis software. Subsequent analysis was carried out in Microsoft Excel applying the delta Ct method.

### Statistical analysis

Overall survival (OS) time was determined as the duration from the start of dosing with MEDI-575 to the date of death. For patients known to be alive at the end of study or lost to follow-up, OS was censored on the last date when patients were known to be alive. Progression-free survival (PFS) was measured from the start of dosing with MEDI-575 to the date of disease progression or death without documented progression. Disease progression is defined according to RECIST guidelines [16]. Progression-free survival was censored on the date of last tumor assessment documenting absence of tumor progression for subjects who had no documented progression and were still alive prior to data cutoff, dropout or the initiation of alternative anticancer therapy. The median OS and median PFS were determined using the Kaplan-Meier non-parametric method [17,18]. The log-rank non-parametric test was used for testing the difference between survivor functions by CTC groups [18].

Stay on treatment time was measured from the date of MEDI-575 first dose administration to the last dose date for enrolled patients. Comparisons of stay on treatment time were made between patients with no, lower, and higher CTC counts using Kruskal-Wallis non-parametric test.

## Results

### Patient Characteristics

A total of 24 patients were enrolled in this Phase I dose escalation trial. Patient characteristics are shown in Table 1. The median age was 65 years (range 39 to 78 years), with 54% of patients being male. The common tumor types in enrolled patients were colon (42%), lung (13%), prostate (13%), breast (4%), ovarian (4%), and endometrial (4%). Nonepithelial malignancies studied included sarcoma and leiomyosarcoma (8%). All patients were at tumor stages III and IV, with predominance of stage IV.

**Table 1:** Patient characteristics.

	Total patients (N=24)	Patients evaluable for CTC enumeration (N=21)
Age (years)		
Median (range)	65 (39-78)	64 (39-78)
Gender		
Male	13 (54.2%)	12 (57.1%)
Female	11 (45.8%)	9 (42.9%)
Tumor category		
Breast cancer	1 (4.2%)	1 (4.8%)
Colon cancer	10 (41.7%)	9 (42.9%)
Endometrial caner	1 (4.2%)	1 (4.8%)
Non-small cell lung caner	3 (12.5%)	3 (14.3%)
Ovarian cancer	1 (4.2%)	1 (4.8%)
Prostate cancer	3 (12.5%)	3 (14.3%)
Others	5 (20.8%)	3 (14.3%)
Tumor stage at study entry		
I–II	0 (0%)	0 (0%)
III	3 (12.5%)	3 (14.3%)
IV	21 (87.5%)	18 (85.7%)
Lymph nodes*		
N0	7 (29.2%)	5 (23.8%)
N+	14 (58.3%)	13 (61.9%)
Metastasis status*		
M0	9 (37.5%)	7 (33.3%)
M+	12 (50.0%)	11 (52.4)

* Information of 3 patients was unknown.

### CTC Enumeration

Among the thirty-three screened patients, 24 were enrolled to the dose escalation portion of the study. Twenty-one blood samples obtained from these 24 enrolled patients at screening (baseline) were evaluable for CTC enumeration. One blood sample was not analyzed because of improper sample shipment, and other two samples from patients with sarcoma and leiomyosarcoma were excluded from analysis as the CTC assay used in this study can only detect tumor cells of epithelial origin. The patient characteristics of the 21 evaluable subjects are shown in Table 1. The CTC count in the 21 evaluable patients ranged from 0-168 per 7.5 ml of blood. Sixty-seven percent (14/21) of the patients showed at least 1 CTC at baseline.

The specificity of the CellSearch® CTC test used in this study has been well tested and validated. In blood samples from healthy subjects, epithelial cells were either not detected or detected in a small percentage (5.5%, 8/145) of subjects at a number of no more than 1 cell per 7.5 ml of blood using the CellSearch® assay [15]. The prognostically relevant cutoff levels of 5, 5 and 3 CTC / 7.5 ml blood have been recommended for breast, prostate, and colorectal cancer, respectively. These cutoff values ensure the assay specificity for CTC detection in cancer patients. Most of current CTC studies have used these cutoff values to classify their subjects into favorable and unfavorable groups for analysis. However, this approach of patient classification neglects the patients with a low number of CTC (under the cutoff levels) by simply grouping them together with patients with 0 CTC. Because this low CTC subpopulation usually constitutes a significant part of the CTC-positive total population, this subpopulation should be analyzed as an independent group. In this study, colon cancer represented 48% of the total patients, so the cutoff level of 3 CTC / 7.5 ml was selected for classifying patients. Subjects were separated into 3 groups, 0 CTC, 1-3 CTC, and greater than 3 CTC, based on their CTC count at baseline. As shown in Table 2, patients with 0, 1-3, and >3 CTC were 33% (7/21) each of all the evaluable patients, respectively.

**Table 2:** Circulating tumor cell detection at baseline.

	**Screened patients**		**Enrolled patients**	
**CTC Number**	**Number of patients**	**% of total patients**	**Number of patients**	**% of total patients**
0	10	34	7	33
1-3	8	28	7	33
>3	11	38	7	33
Total	29	100	21	100

### Association between baseline CTC counts and stay on treatment time

**Figure 1:**
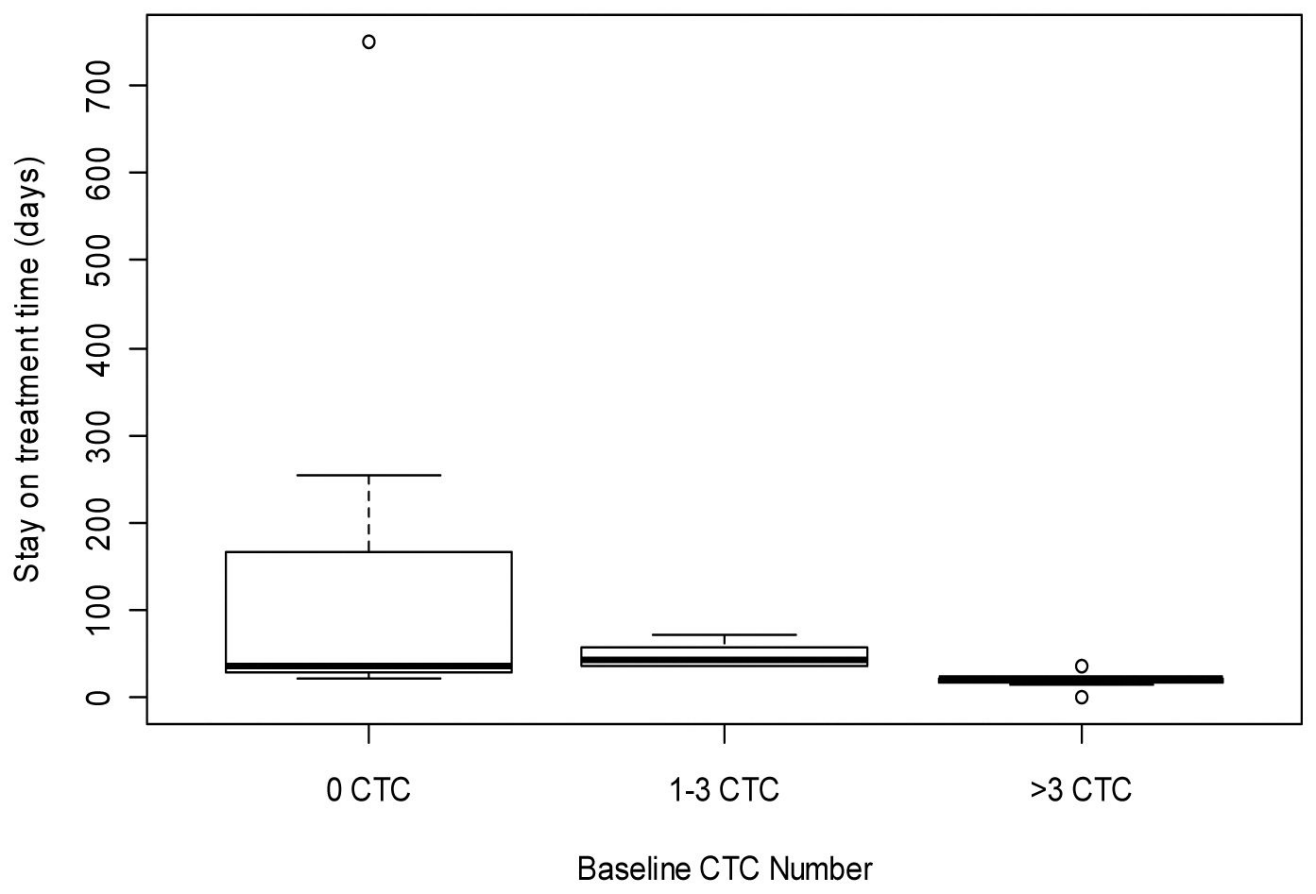
Circulating tumor cell detection and patient stay on treatment time.

Stay on treatment time (treatment duration) was compared between patient groups with 0, 1-3, and >3 CTC (Figure 1). Patients with >3 CTC at baseline tend to have a shorter stay on treatment time (mean=20 days; median=22 days) than patients with 1-3 CTC (mean=48 days; median=43 days, *P* < 0.05) and patients with 0 CTC (mean=171; median=36 days, *P* < 0.05).

### Association between baseline CTC counts and survival

**Figure 2:**
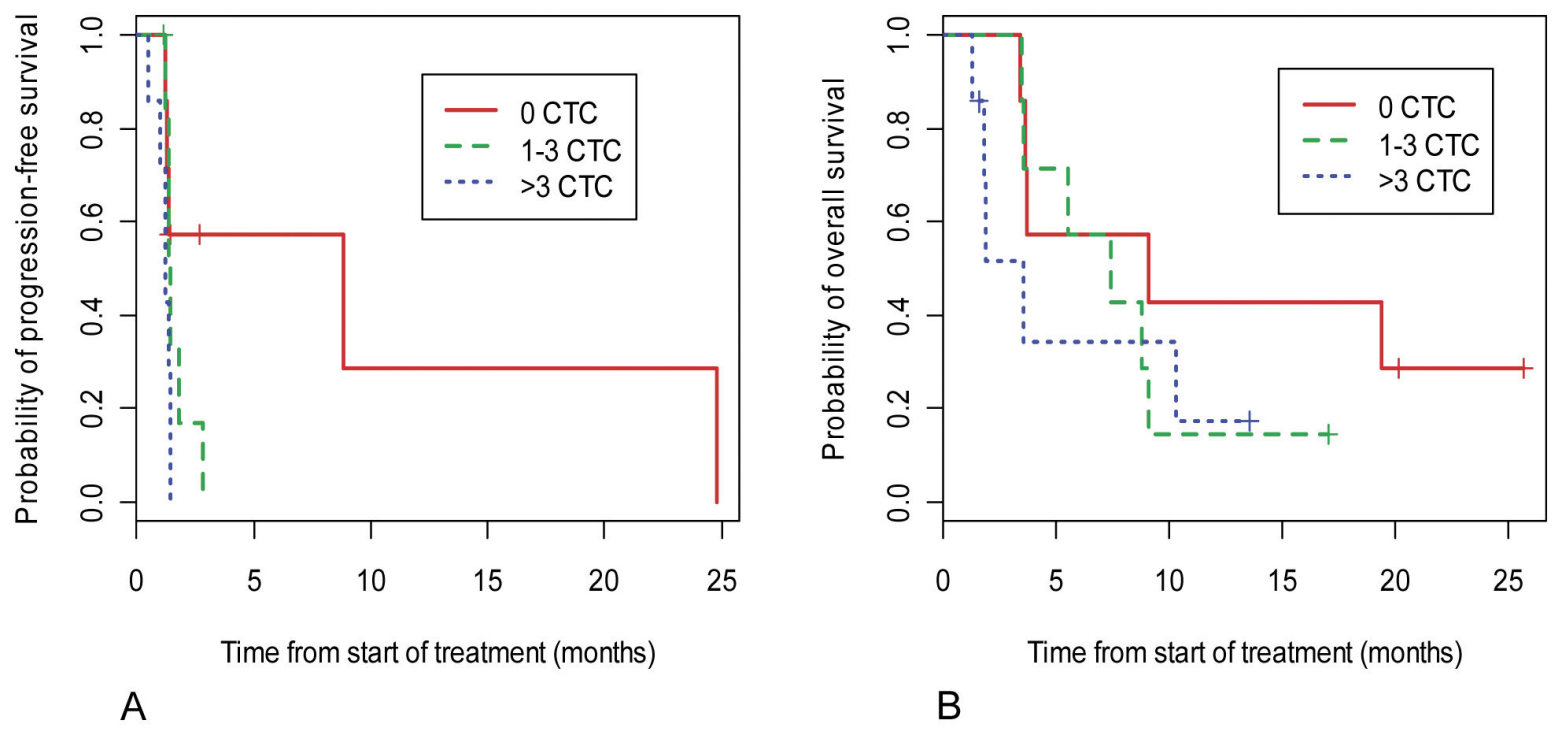
Kaplan-Meier estimates of probabilities of overall survival (A) and progression-free survival (B) of patients with respect to frequency of CTCs before initiation of therapy. The red, green, and blue lines represent patients with no CTC, 1-3 CTCs, and above 3 CTCs, respectively.

The survival analysis was based on the study data as of June 22, 2011. At that time there were 16 deaths out of 21 patients analyzed. A Kaplan-Meier plot comparing PFS for patient groups with different CTC counts at baseline is presented in Figure 2A. The PFS medians were 8.8, 1.4 and 1.3 months in patient groups with 0 CTC, 1-3 CTC, and >3 CTC, respectively. A *P*-value of 0.02 was obtained from the log-rank test for the difference between the PFS curves of the three CTC groups. Figure 2A also shows that the difference primarily existed between patients with 0 CTC and patients with one or more CTC, with little difference between patients with 1-3 CTC and those with > 3CTCs.

Figure 2B shows Kaplan-Meier curves for OS in the enrolled patients according to their CTC count at baseline. The median OS of the patients with 0 CTC was 9.0 months, compared with an OS of 7.4 and 3.5 months for the groups with 1-3 and greater than 3 CTC, respectively. The log-rank test for testing the survival difference among the three groups gave a *P* = 0.20.

### CTC counts and treatment response

**Figure 3:**
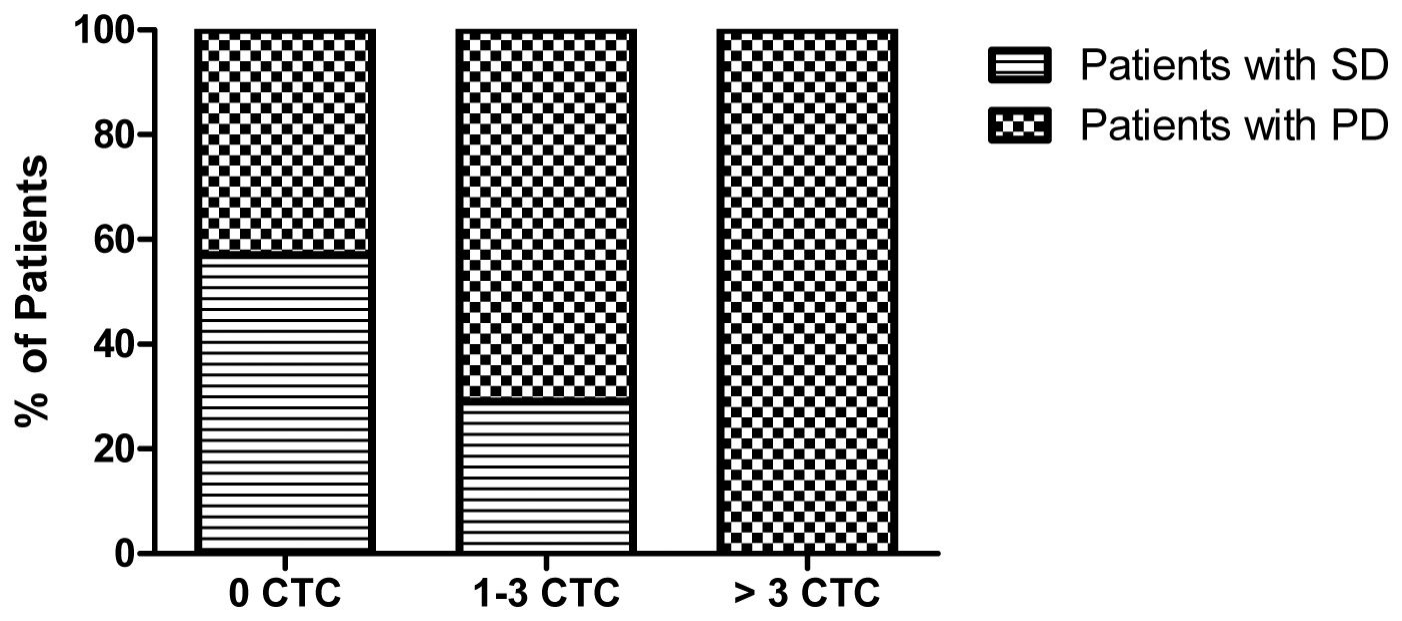
Circulating tumor cell number and disease outcome.

Figure 3 summarizes the disease status of 21 evaluable patients of the total 24 enrolled patients based on their radiographic response and CTC counts at baseline. All (7/7) of the patients with >3 CTC showed progressive disease (PD). Of patients with 1-3 CTC, 29% (2/7) had stable disease (SD) and 71% (5/7) had PD. In comparison, 57% (4/7) of patients with 0 CTC had SD and 43% (3/7) had PD.

Effect of MEDI-575 on CTC count could not be assessed because a number of patients, especially those with >3 CTC, only finished 1 cycle of treatment. Collection of blood samples at the start of subsequent cycles for CTC evaluation could not be completed. As a result, too few pairs of CTC counts pre- and post-treatment were available for analysis considering the patient sample size in this study was already small.

### Characterization of CTC with the multimarker qRT-PCR assay

A multimarker qRT-PCR assay was developed for gene expression profiling of CTC samples collected at screening in 15 patients. The development and characterization of this assay has been previously reported [19] and a separate manuscript is being prepared and will be reported elsewhere. The multimarker qRT-PCR assay used in this study included 8 genes: CK20, CEA, AGR2, MGB2, DLL4, EphA2, Her3, and PDGFRα. During the assay development, all the above genes were confirmed as not being expressed in leukocytes from healthy donors. Of the 8 genes, CK20, CEA, AGR2, and MGB2 are epithelial / tumor markers that have been used previously to detect CTC by RT-PCR [20–22]. PDGFRα, the drug target of MEDI-575, and the other 3 genes, DLL4, EphA2, Her3, served as exploratory genes in this study. These exploratory genes are associated with cancer and are being investigated as potential drug targets for treatment of cancer. The main purpose of the assay was to assess expression of the drug target genes in CTC-enriched samples, and detection of epithelial / tumor markers in the same samples further verified their connection to CTC.

House-keeping genes, including 18S, β-actin, and GAPDH, were detected in all 15 samples by the multimarker qRT-PCR assay, indicating RNA extraction from CTC enriched samples was adequate. Samples from 6 of 15 patients (40%) showed expression for at least one of the 3 CTC markers, CK20, CEA, AGR2 (Table 3). Expression of the mammaglobin gene MGB2 was not detected in any patient sample, which is consistent with the result that the only breast cancer patient in this study had 0 CTC by the CellSearch® assay. Expression of Her3, EphA2, and DLL4 (exploratory genes) was found in 3, 4, and 3 patients, respectively. PDGFRα expression was not detected in any patients by the multimarker qRT-PCR assay.

**Table 3:** Circulating tumor cell gene expression profiles by quantitative RT-PCR.

**CTC count**	**House-keeping genes**			**Tumor markers**				**Exploratory genes**			
	**18S**	**β-actin**	**GAPDH**	**CECEAM5**	**KRT20**	**ARG2**	**MGB2**	**HER3**	**DLL4**	**EPHA2**	**PDGFRα**
168	+++	+++	+++	++	++	+	-	+	-	++	-
79	+++	+++	+++	-	-	-	-	-	-	-	-
74	+++	+++	+++	-	-	++	-	++	++	+	-
48	+++	+++	+++	-	-	-	-	-	-	-	-
19	+++	+++	+++	-	-	-	-	-	-	-	-
11	+++	++	++	+	-	-	-	-	-	-	-
2	+++	++	++	++	-	-	-	-	-	+	-
2	+++	+++	+++	-	-	-	-	-	-	-	-
1	+++	++	++	-	-	-	-	-	+	-	-
1	+++	++	+++	-	-	-	-	-	-	-	-
1	+++	+++	+++	+	-	-	-	-	-	-	-
0	+++	+++	+++	++	+	-	-	-	+	+	-
0	+++	++	++	-	-	-	-	-	-	-	-
0	+++	+++	+++	-	-	-	-	+	-	-	-
0	+++	+++	+++	-	-	-	-	-	-	-	-

+++ <20 CT

++ 20-25 CT

+ 25.1-29 CT

- Undetected

The concordance of CTC detection by the CellSearch CTC test and the multimarker qRT-PCR assay was low. Agreement between the 2 detection methods was observed in only 47% of samples. A significant difference in the detection rate of CTC by the CellSearch® and RT-PCR methods has been reported by others [22]. The cause of this discrepancy is unclear but is at least partially due to different markers being used for CTC identification in these two methods. The original design of the multimarker qRT-PCR assay included more numbers of widely expressed epithelial / tumor markers in order to cover various tumor types expected in the all-comer clinical trial. During the assay development, however, we found that some of these widely expressed epithelial / tumor markers, such as EpCAM, HER2, and CK19, were detectable in blood samples from some healthy donors by the very sensitive qRT-PCR assay. To ensure the specificity of CTC detection, these markers were excluded from the multimarker qRT-PCR assay used in this study. The CTC detection rate, therefore, could be underestimated by the multimarker qRT-PCR assay.

## Discussion

CTC may represent a rich source for potential biomarkers that can be used to assist drug development, such as early prediction of therapeutic efficacy and enrichment of patients most likely to respond to a given targeted therapy. To our knowledge, this study is the first report of incorporation of CTC analysis, including both enumeration and molecular characterization using gene expression profiling, into an all-comer solid tumor Phase I clinical study of an investigational agent. Twenty-one patients were eligible for CTC enumeration in this study. Sixty-seven percent (14/21) showed at least 1 CTC at baseline (patient screening). Despite the small number of patients and heterogeneous cancer types, this study demonstrated consistent data showing that the CTC count at baseline was a strong predictor of patient clinical outcomes. Patients with 0 CTC at baseline had a longer PFS and OS than those with 1-3 CTC and >3 CTC at baseline (Figure 2). The patients with 0 CTC at baseline also showed more time on study than those patients with 1-3 CTC or >3 CTC at baseline (Figure 1).

In addition to the small patient sample size, another challenge for incorporating a CTC biomarker analysis into a Phase I clinical study is having a variety of tumor types in the study. In this study, colon, lung, prostate, ovarian, breast, and endometrial cancer accounted for 78% of the total patients. The CellSearch® CTC test has currently been approved by FDA for detection of CTC in patients with breast, prostate and colorectal cancer to monitor disease progression. This test has also been reported to measure CTC from various other solid tumors such as lung, pancreatic, and ovarian [2]. The CTC detection rate may vary by tumor types, disease stages, and metastatic status. Using the CellSearch® system, Allard et al. showed an average detection rate of 36% of patients with ≥ 2 CTCs per 7.5 ml of blood from a total of 2183 patient samples, including a detection rate of 57% for prostate cancer, 37% for breast cancer, 37% for ovarian cancer, 30% for colorectal cancer, 20% for lung cancer, and 26% for other cancer [2]. The recommended cutoff number of the CellSearch® CTC test for metastatic breast and prostate cancer is 5 CTC and for metastatic colorectal cancer is 3 CTC. The cutoff number for other types of solid tumor has not been determined for the CellSearch® system. After reanalyzing the data from the multicenter trials of metastatic breast cancer, Tibbe et al. reported that the presence of even 1 CTC in 7.5 ml of blood detected by the CellSearch® CTC test has clinical relevance with regards to disease outcome [23]. Bidard and coworkers demonstrated detection of a single CTC in 7.5 ml of blood was associated with poor OS and development of metastasis in breast cancer patients [24]. Olmos et al. showed that higher CTC count as a continuous variable was correlated with patient death in Phase I trials with various tumor types [25]. We separated the patients with detectable CTC into 2 groups (1-3 and >3 CTC) based on their CTC number at baseline. Both groups, 1-3 and > 3 CTC, had significantly worse outcomes in PSF, OS, and radiographic response as compared to the patients with 0 CTC at baseline, although the group with >3 CTC tends to show the worst outcome (Figure 2, Table 2). Our findings agree with the view that detection of CTC even at low numbers (<3 CTC) by the CellSearch® CTC test may have clinical relevance. Therefore, the patients with a CTC number under the cutoff level should be considered as an independent group for analysis, instead of being grouped together with patients with 0 CTC. The quantitative relationship between CTC count and patient survival needs to be further investigated in studies with a larger number of patients.

The observations from this trial also suggest that CTC may be useful as a stratification biomarker in future Phase I oncology clinical studies to assess clinical response of investigational agents and to better inform early go / no go decision making. Due to the poor prognosis of the patient, Phase I studies often evaluate clinical response of an investigational agent by assessing stable disease after 3 or 4 treatment cycles or greater than 100 days. Stratification of patients into the 3 categories described in this study may be useful for assessing clinical response of an investigational agent by minimizing the potential confounding prognostic factors. Further studies are needed to evaluate whether this adds additional information in assessing clinical benefit of new agents.

In this study, we evaluated the relationship between the baseline CTC count and stay on treatment time. Our results demonstrated that patients with no CTC or a lower CTC count had a significantly longer stay-on treatment time than those with a higher CTC count. This observation indicates that patient stratification based on the CTC number may be useful for clinical trials with special features such as cancer immunotherapy trials. Different from conventional chemotherapies, immunotherapies often show delayed clinical effects and need a longer treatment and observation period [26]. Patient loss before the time window of clinical effects impedes assessment of therapeutic activities especially in early clinical trials with patients with advanced cancer. Therefore, selection of patients with no CTC or a lower CTC count may reduce the patient loss and warrant a proper evaluation of investigational immunotherapeutic agents in early phase clinical trials. It has been reported that incorporation of the CTC count could improve the performance of prognostic score used for patient selection for phase I trials [25].

Results from previous studies demonstrated that the decrease in CTC counts following a therapy was predictive of the effectiveness of the treatment in a specific indication [6–8]. The dose-effect relationship between MEDI-575 and CTC count could not be determined in our study because there were insufficient pairs of specimen from pre- and post-treatment available for a quantitative analysis. We did not have post-treatment blood samples from those patients who only received 1 cycle of treatment. This is a major limitation of this study. A blood sample collection at the end of study needs to be implemented in order to secure post-treatment data and has been incorporated in all new studies.

Gene expression profiling of CTC has not been widely explored because of technical limitations such as low cell numbers, cell fixation, and extensive leukocyte contamination. The expression of drug target in tumor cells can be a predictive biomarker of response to a molecularly targeted therapy such as transtuzumab. Furthermore, transtuzumab ineligible patients based on diagnostic biopsy samples have been tested for Her2 expression on CTC and CTC Her2 positive patients have shown benefit to transtuzumab treatment [9]. A multimarker qRT-PCR assay was developed in this study to explore expression of selected genes, especially the drug target PDGFRα, in CTC from patients with various types of solid tumors. The multimarker qRT-PCR assay consisted of 2 groups of genes: 4 epithelial / tumor markers to identify CTC and 4 cancer-associated genes for exploration. All 8 genes were confirmed as not being expressed in leukocytes before the study. CTC markers were found in 40% (6/15) of patients by this assay (positive by at least one of the 4 epithelial/tumor markers) (Table 3). The concordance between detection of 3 exploratory genes (except PDGFRα) and tumor markers in patient samples was 80% (8/10) using the multimarker qRT-PCR assay. DLL4, a predominantly endothelial specific gene, has also been reported to be expressed on cancer stem cells [27]. The prevalence of detection of DLL4 expression was a surprise in this study. Immunohistochemistry analysis of multitumor microarrays has shown that tumoral expression of PDGFRα is detected in NSCLC, colorectal, and ovarian cancer (data not shown). The unexpected result was that PDGFRα expression in CTC was not detected in any of the patients evaluated. The same result was also observed in cancer patient samples purchased from vendors (data not shown). Sieuwerts et al. reported that CTC that undergo epithelial-mesenchymal-transition (EMT) do not express or express a low level of EpCAM, and these epithelial-mesenchymal transition cells could not be detected by EpCAM-dependent CTC assays [28]. The multimarker qRT-PCR assay in this study used CTC samples enriched by anti-EpCAM antibody-coated magnetic beads. This led to our investigation of co-expression of PDGFRα and EpCAM using cancer cell lines. Our results revealed that the expression of PDGFRα and EpCAM was mutually exclusive in all cancer cell lines tested (data not shown). MEDI-575 is currently in a Phase Ib/II clinical study in NSCLC (http://clinicaltrials.gov/ct2/show/NCT01268059) in which CTCs will be analyzed and archival tumor specimens will be assessed for PDGFR-α expression.

In conclusion, this study reports for the first time the feasibility of performing a CTC biomarker study, including both enumeration and gene expression profiling, in an all-comer solid tumor Phase I clinical trial. Measurement and molecular characterization of CTC has great potential as a biomarker to gain disease and treatment information from patients to support decision making in drug development.

## Supporting Information

Figure S1 **Flowchart of participants through the CTC study.**
(TIF)
