# Supplementary figures and images for: Circulating Tumor Cells: Application as a Biomarker for Molecular Characterization and Predictor of Survival in an All-Comer Solid Tumor Phase I Clinical Study
Source: PLoS One. 2013 Aug 21;8(8):e58557. doi: 10.1371/journal.pone.0058557 (PMC3749129; doi:10.1371/journal.pone.0058557)

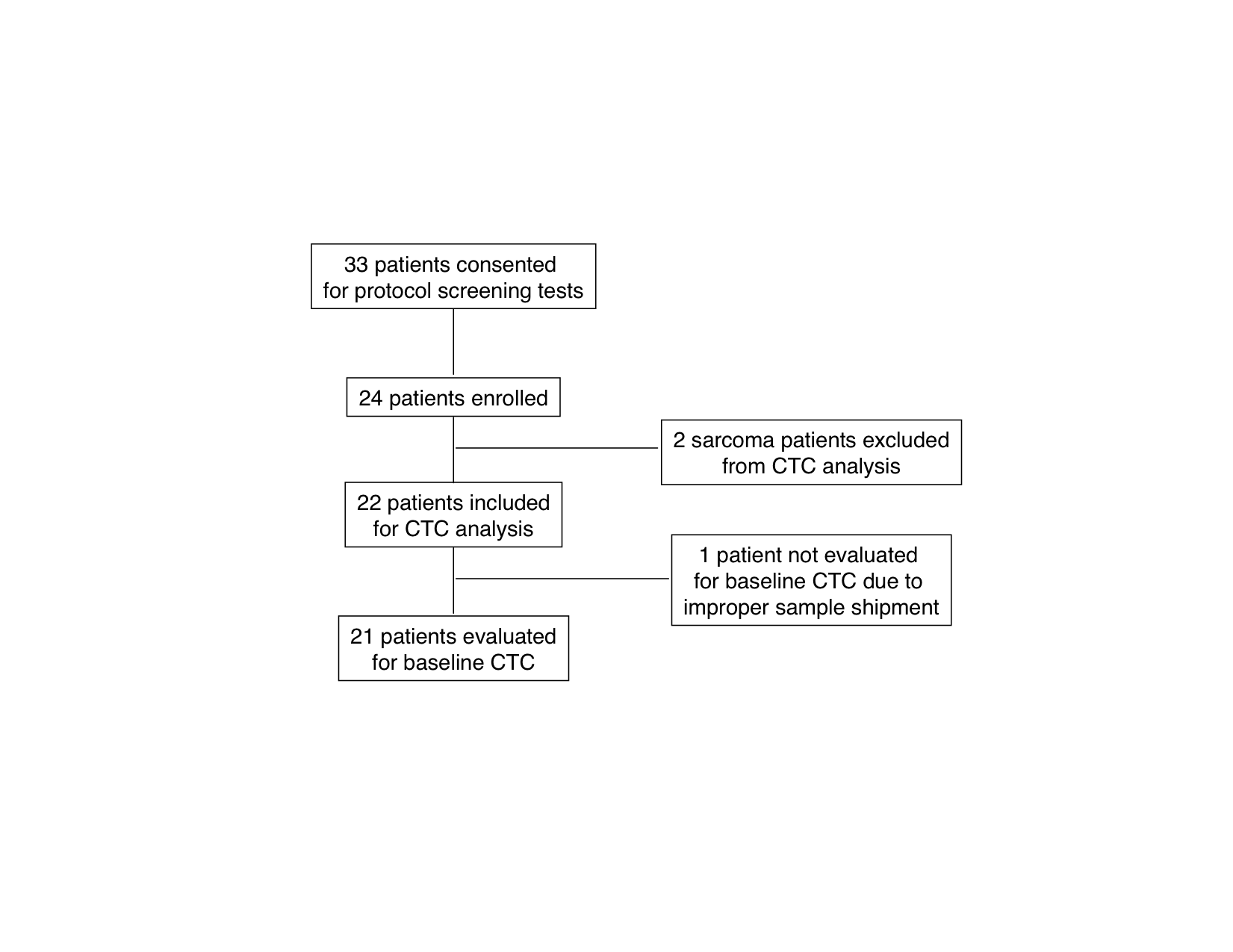

Supplement: Figure S1 — Flowchart of participants through the CTC study. (TIF) [file pone.0058557.s001.tif]
